# Effects of bi-hemispheric anodal transcranial direct current stimulation on soccer player performance: a triple-blinded, controlled, and randomized study

**DOI:** 10.3389/fspor.2024.1350660

**Published:** 2024-03-22

**Authors:** Jader Vinicius Da Silva Rocha, Rodrigo Freire de Almeida, Bárbara Naeme de Lima Cordeiro, Carlos Henrique Cardoso Sarcinelli, Carla Zimerer, Fernando Zanela Arêas

**Affiliations:** ^1^ Universidade Federal do Espirito Santo, Vitória, Brazil; ^2^ Department of Physiological Sciences, Federal University of Espirito Santo, Vitória, Brazil

**Keywords:** neuromodulation, neurophysiology, electrical stimulation, tDCS, performance, football, soccer

## Abstract

The search for increased performance and physical performance are linked to the use of ergogenic resources. The vertical jump is one of the measures commonly used to evaluate the performance of lower limbs in athletes. Transcranial direct current stimulation (tDCS) is a non-invasive, safe, economically viable technique that can modulate cortical excitability, which can influence the increase in the performance of athletes in general. This study aimed to investigate whether the use of tDCS on the primary motor cortex (M1) improves the performance of soccer players. A cross-sectional study was conducted. Twenty-seven players were randomized into three groups: Active tDCS group (*n* = 9), Sham group (*n* = 9), and control group (*n* = 9). Stimulation was applied at 2 mA for 15 min using a cephalic mount. Visual Pain Scale (VAS) and Subjective Recovery Scale (SRS) were monitored before and after tDCS. In addition, the participants performed the Countermovement Jump (CMJ) before and after the stimulation intercalated with Heart Rate (HR) and Rating of Perceived Exertion (RPE CR-10). No differences were found in any of the performance variables analyzed (*p* > 0.05) nor in the responses of HR (*p* > 0.05), RPE (*p* > 0.05), VAS (*p* > 0.05), and SRS (*p* > 0.05) between groups. The tDCS in M1 did not change the performance of the vertical jump, and there was no improvement in the subjective scales. New studies should also be developed with different stimulus intensities in different cortical areas and sports modalities.

## Introduction

During the competition season, athletes need to seek to improve their performance. In this sense, training loads (i.e., frequency, duration, and intensity) become high (1). Therefore, athletes may experience symptoms of fatigue that decrease muscle capacity for performance (1, 2). Consequently, controlling the training load throughout the season is essential to evaluate performance and avoid fatigue-related problems, such as non-functional overreaching (fatigue lasting from weeks to months), injuries, and illnesses (1).

One of the methods to monitor training load and recovery is the countermovement jump (CMJ) (1, 3, 4). The CMJ test assesses neuromuscular function through lower body power, quantified through jump height (i.e., power). During CMJ, power can be measured using various tools such as contact mats, force platforms, infrared platforms, accelerometers, linear position transducers, and/or video analysis (5, 6). Thus, the participant is asked to squat quickly for a self-selected downward action, followed by a reciprocal upward action, jumping as high as possible (5, 7). The CMJ assessment may indicate a decrease or increase in performance since the rate of strength development analyzed is related to muscle strength. Therefore, the greater the height of the CMJ, the more muscle force is used being an indication of low muscle fatigue (8).

Exercise induced muscle fatigue involves processes at various level of the motor pathway, from the brain to the muscle (9). These processes involve reductions in motor cortex excitability (10, 11), spinal excitability (12, 13), and in the contractile capacity of the recruited muscle fibers (14).

Currently, several ergogenic strategies are used to optimize sports performance. These strategies are beneficial ergogenic measures for recovery and performance, such as rest, adequate sleep, hydration, physiotherapeutic resources, nutrition, and neurostimulation techniques (15, 16). In addition, ergogenic measures that aim to increase supraspinal excitability can lead to a more efficient motor command that, ultimately, could increase the time in which a fixed output could be maintained (for example: muscle power benefit). This hypothesis is already being tested in some sports and several studies have shown improvement in a neuromodulatory technique called transcranial direct current stimulation (tDCS) (17–20). Neurostimulation techniques have been proposed to improve athletes cognitive and psychomotor performance. Among them, non-invasive brain stimulation, such as transcranial direct current stimulation (tDCS), is becoming famous for improving sports performance. The reason is the safety, low-cost, and easy-to-apply technique (19). The use of tDCS consists of applying two electrodes with a low-intensity continuous current (1–2 mA) in specific areas of the brain for a particular time (7–25 min) (21). This stimulation appears to induce changes in cortical excitability lasting from minutes to several hours after its use (22).

Among the areas that can be stimulated by tDCS, the primary motor cortex (M1) stands out as the brain region most related to sports performance due to its role in driving the exercised muscles (19). Research has shown that core fatigue can impact overall exercise performance. In this context, the decrease in motor neuron excitability and the limited ability of M1 and other supraspinal areas to maintain or increase the neural impulse may decrease the muscular capacity to produce force/power, thus leading to fatigue. It is known a single anodal tDCS session in M1 can lead to performance enhancement in athletes in sport-specific motor tasks (17).

However, the literature indicates that results about the effects of tDCS on jumping performance are controversial. For example, Lattari et al. (23) observed an increase in the vertical jump performance of young men with experience in strength training from 20 min of tDCS in the primary motor cortex. On the other hand, Arenas et al. (24), found no significant improvement in vertical jump performance with 15 min of tDCS in the left dorsum lateral prefrontal cortex of healthy young non-athletes. These results may have occurred due to differences in the study methods, such as voltage, stimulation time, target brain area stimulated, or studied population.

Also, as indicated in the literature, studies investigating the effect of tDCS on the performance of soccer players are scarce and necessary (25, 26). These surveys, primarily carried out during the competition period, can help coaches and soccer players control the training and recovery load. In this context, the present study aimed to evaluate the effect of 15 min of anodic tDCS at M1 on vertical jump performance in young soccer players. Based on the available evidence regarding the role of tDCS on performance in athletes, the hypothesis of this study is that tDCS will increase vertical jump performance and improve perceived exertion and recovery compared to sham tDCS.

## Methods

### Subjects

The sample size was calculated using the G*Power software (version 3.1) (27–29) based on the prior analysis method. For analysis, the following commands were used: test family 5 *F*-tests, statistical test 5 analysis of variance: MANOVA repeated measures within-between interaction, error probability *α* 0.05 and statistical power of 0.96. Effect size was defined as a moderate effect size of 0.25, sphericity *ε* = 1. The sample size was determined at 27 individuals. The 27 soccer players of the U20 team (age: 18 ± 0.77, body mass: 73.8 ± 6.50 kg, height: 178 cm ± 8.44 cm, body fat: 8.7 ± 2.55%, BMI: 23.30 ± 1.9 kg/m^2^).

Subjects were randomized one of the conditions experimental: (1) anodic tDCS over the motor cortex, (2) sham tDCS and (3) control group. The player-specific training schedule consisted of 5–6 weekly sessions, each lasting −90–120 min, and one or two competitive games per week. The inclusion criteria for participation contain the application of the Physical Activity Readiness Questionnaire (PAR-Q+) (30). Players between 18 and 20 years old without injuries and available to play. The exclusion criteria include any orthopedic injuries and/or mental health problems (e.g., schizophrenia) and/or brain disorders (e.g., epilepsy), intracranial implants, and using any psychoactive medication during the study. Before signing the written informed consent, the participants were informed about the procedures and possible risks. The research was approved by the local Research Ethics Committee (approval number: 40396120.6.1001.5106) under the Declaration of Helsinki.

## Study design

**Figure 1:**
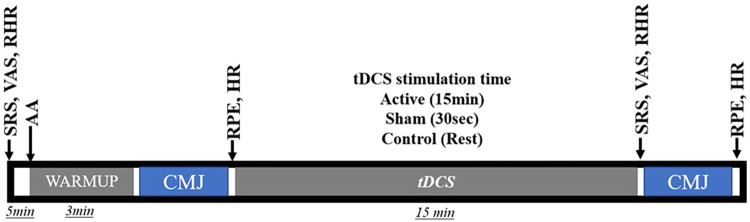
Study flow diagram; RHR, resting heart rate; SRS, subjective recovery scale; RVAS, resting visual analogue scale; CMJ, countermovement jump; RPE, rating of perceived exertion; HRP, rate heart post CMJ; VASP, visual analogue scale post CMJ.

Participants made four visits, one preliminary and three experimental sessions. All experimental sessions were separated by 24 h. Visits were simultaneously on the experimental days in a temperature-controlled room (−24°C). Participants were instructed to keep the same diet except for drinking coffee or energy drinks during the test week. On the first visit, they were familiarized with all procedures of all three sessions, which included, respectively, (a) the assessment of the Subjective Recovery Scale (SRS) and Visual Analogue Scale (VAS), (b) after 5 min seated the measurement of Rest Heart Rate (RHR); (c) the warmup which includes 1× with 10 s of interval of the following exercises: reverse lunge (20 reps), walking holding the knee unilaterally (20 reps for 3 m), isometric squat (for 15 s) with arms extended in front, low and medium skipping (20 reps). Warmup estimated time (3 min); (d) 3reps of CMJ followed by the Rating of Perceived Exertion (RPE) scale (CR-10) and the Rate Heart (HR) assessment; (e) After this, they did the *Intervention*, which means the adjustment of the tDCS apparatus and the stimulus for each group [for the active group (15 min of stimulus), sham (30 s of stimulus) and control (rest for 15 min, with no stimulus and without device)]; (f) The SRS, VAS and RHR; (g) 3 reps of CMJ; (h) RPE, and HR assessment. All data included all participants who completed the four sessions' experimental procedures (Figure 1). During the procedures, unique in the CMJ, all participants were stimulated to do their best. The study design is a triple-blinded, controlled, and randomized fashion.

### Allocation

Randomization results were generated using a computer-generated random number sequence by an outsider researcher who was not involved in allocating or assessing study participants. Subjects were randomized one of the conditions experimental: (1) anodic tDCS over the motor cortex, (2) sham tDCS and (3) control group. A second researcher opened randomly ordered, consecutively numbered envelopes containing the results of group allocation after the initial assessment.

### Blinding

In the active and sham tDCS conditions, researchers and participants were blinded by an independent researcher who did not participate in the other stages of the study. However, during the controlled condition, it was not possible to maintain blinding, as this condition did not require the use of equipment. The choice to carry out a control condition was determined with the intention of ensuring that at least one intervention avoided any possible psychological effects (placebo) generated using the equipment.

### Transcranial direct current stimulation procedures

**Figure 2:**
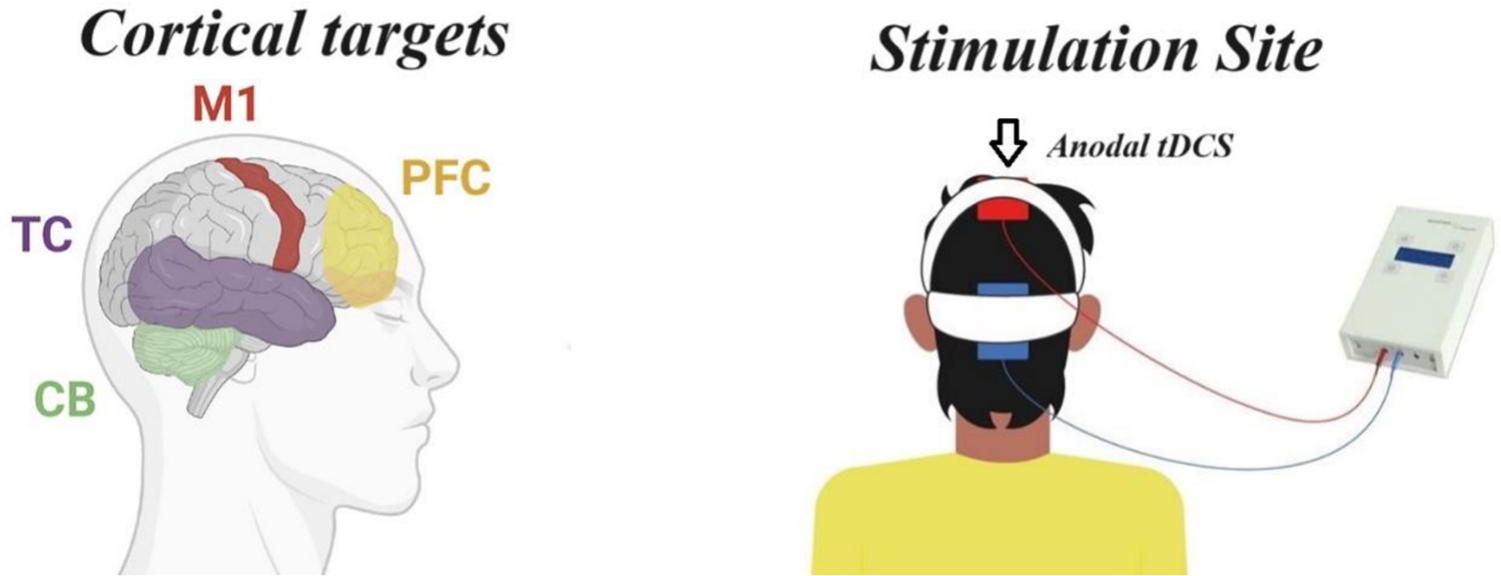
tDCS position.

Consistent with previous research, the study targeted the primary motor cortex (M1). For the anodal condition (a-tDCS), the electrodes were positioned to target the M1 bilaterally points CZ according to the international 10–20 EEG system (21, 25, 31, 32). Anodal tDCS over M1 facilitates motor performance and learning (33, 34), most likely by eliciting long-lasting, polarity-dependent changes in cortex excitability (22, 35). While the cathodal electrodes were placed on the inion (Figure 2) (36). Is possible to the tDCS montage influence other brain areas related to performance, such as parietal cortex or occipital. A more specific stimulation should be applied to investigate each area separately (36).

The intensity and duration of tDCS were based on previous studies (19, 20, 36). tDCS might strengthen synaptic connections through a mechanism that is like long-term potentiation (LTP), a cellular mechanism that underlies memory consolidation and learning (37, 38). The tDCS was administered using a battery-powered stimulator (Neuroconn, Ilmenau, Germany) through a pair of rubber electrodes (size: 6 cm × 8 cm, 48 cm^2^) wrapped in a sponge soaked in saline liquid (9% NaCl). The electrodes were fixed to the head by elastic straps. Stimulation intensity was set at 2 mA for a period of 15 min. In both experimental conditions (active or sham), the amplitude of the electrical current was progressively increased and decreased over the first and last 30 s of the session, and in the sham group, the current was interrupted after this initial period. Activation of the transcranial direct current stimulation (tDCS) device occurred using a code provided by an external researcher, responsible for correcting the blinding. The automatic shutdown or not of the equipment (active or sham) was programmed using stimulation codes (active or sham) kept confidential, using the program in research mode of the mobile stimulator (DC-Stimulator, Neuroconn Mobile tDCS). This program provided a list of codes provided for the allocation of participants in relation to the active or simulated stimulation condition. Electrical resistance was maintained between 4 and 6 kΩ. In the control condition, participants rested quietly for 15 min. A total of 3 consecutive sessions were performed on different days in the afternoon with a 24-hour interval between sessions. At the end of each session, participants were asked to answer a questionnaire to assess any adverse effects, and no adverse effect was pointed out except for itching and tingling sensation under the electrodes during tDCS.

### Performance assessments

In each experimental session, several measurements were taken, including HR, VAS, SRS, and RPE, to assess the physiological and subjective responses. Additionally, the jump height and power evaluation during the CMJ was conducted at two specific time points: after the warmup (Pre) and after 15 min of stimulation/sham/control (Post).

### Heart rate (HR)

Each rest HR measurement was taken before the warmup, immediately after stimulation, respectively, after five and 15 min of rest. Also, the HR was taken after the first and second CMJ. This variable was used to get the participant's effort measuring the internal load (1, 39).

### Visual analog scale (VAS)

In each experimental session, VAS measurements were conducted at two time points: at rest and immediately after the Intervention (to the active and sham group) or after the rest to the control group. The VAS is a prevalent instrument for pain measurement (40).

### Subjective recovery scale (SRS)

The SRS was taken in 2 moments, in rest condition, immediately after Intervention (to the active and sham group) or after the rest to the control group. This variable shows whether the participants recovered from the previous game and/or session. Furthermore, this is essential in determining the participant's physical status and readiness for the next session (41). This subjective approach may be practical for assessing recovery daily under similar conditions (42).

### Countermovement jump (CMJ)

**Figure 3:**
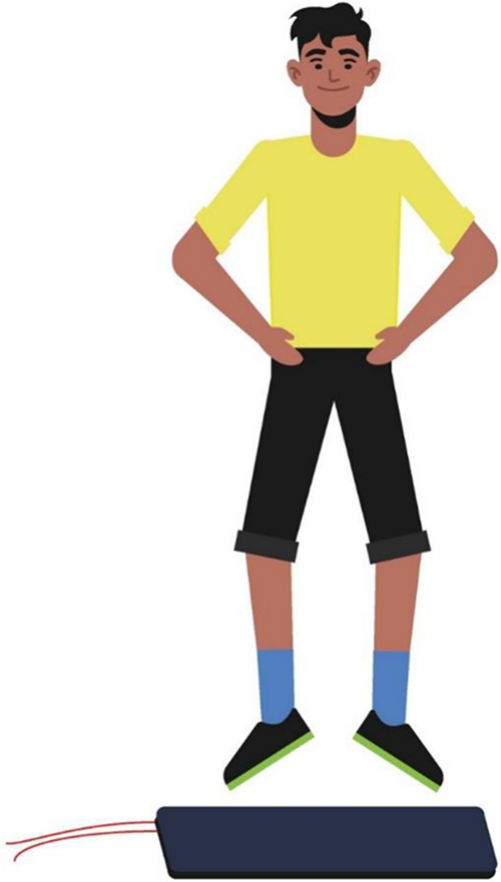
Countermovement jump.

Before the first CMJ, the participants did the warmup protocol, Afterward, each group followed their procedures, according to the Intervention described before, and performed the CMJ again. Each CMJ was performed with three consecutive jumps, which involved three consecutive jumps (adaptation of the Abalakov protocol) (43). CMJ was performed with the subject standing in a with the hand on the hips to avoid arm swings. A fast downward movement was immediately followed by a fast upward vertical movement as high as possible, all in one sequence (Figure 3). The jumps were executed on a Jumptest® platform (Hidrofit Ltda, Brazil) measuring 50 cm × 60 cm, connected to the Multisprint® software (Hidrofit Ltda, Brazil) (44). The average CMJ data provided by the software, according to Claudino et al. (45), were used in the results. The variables assessed were the Jump Height (CMJ-JH), Power (CMJ-PO). Based on meta-analysis (5), the average CMJ height is more sensitive than the highest CMJ height in detecting fatigue and CMJ overcompensation. Furthermore, other CMJ variables, such as power, average power, peak velocity, peak force, average impulse and power, were evaluated better at the average height compared to the maximum height of the CMJ.

### Rating of perceived exertion borg (RPE-CR-10)

After each CMJ, the participants provided their RPE from 0 (noticeable) to 10 (max), according to Foster et al. (39). Then they proceeded to their respective groups at the (active/sham or control). After a 15-minute interval, they performed the CMJ again, followed by the RPE. The RPE method can estimate exercise load, including high-intensity interval training, team sport practice, and competition (39).

### Statistical analysis

The normality assumptions of the data were checked using the Shapiro-Wilk test. MANOVA test was performed for all variables using a significance level of *p* < 0.05. All the assumptions of MANOVA were checked, such as multivariate normality, absence of multivariate outliers, absence of multicollinearity, linear relationship between dependent variables at each level of independent variables, homogeneity of variance-covariance matrices, and independent observations. The partial eta-squared (*ηp*^2^) was used as the effect size measure (classified as small: 0.01, moderate: 0.09, large: 0.25). A two-way ANOVA was used to generate statistical interaction (time × groups) and repeated one-way ANOVA was used to test between and within-group differences throughout the protocol variables, which is included the anthropometric measurement differences determination. The Tukey test was used as a *post hoc* analysis for the MANOVA procedures and Bonferroni to the ANOVA procedures.

The analyses used the Statistical Package for the Social Sciences (SPSS) version 21. GraphPad Prism software version 8.0 (GraphPad Software, Inc., San Diego, CA, USA) generated graphs.

## Results

The results of the Rest Heart Rate (RHR) are described in Table 1. The MANOVA results [Wilks' Lambda (*W*) = 0.446, *F* (2.24) = 0.537, *p* = 0.934, effect size (*ηp*^2^) = 0.331] did not detect differences between groups for RHR before the first CMJ. The absence of difference persists at the univariate ANOVA in all three days within the groups, respectively RHR day 1 (RHRD1) [*F* (2.24) = 0.074, *p* = 0.929, *ηp*^2^ = 0.006], RHRD2 [*F* (2.24) = 0.295, *p* = 0.747, *ηp*^2^ = 0.024], and RHRD3 [*F* (2.24) = 0.230, *p* = 0.796, *ηp*^2^ = 0.019].

**Table 1:** HR, heart rate; RHR, resting heart rate; PHR, post-intervention rate heart; pre-intervention (pre); post-intervention (post); confidence interval in 95% (CI 95%); M, mean; SD, standard deviation; IL, inferior limit; SL, superior limit.

HR	Group	Pré day 1			Post day 1			Pré day 2			Post day 2			Pré day 3			Post day 3		
		M ± SD	CI (95%)		M ± SD	CI (95%)		M ± SD	CI (95%)		M ± SD	CI (95%)		M ± SD	CI (95%)		M ± SD	CI (95%)	
			IL	SL		IL	SL		IL	SL		IL	SL		IL	SL		IL	SL
HRR	INT	62.5 ± 3	55.6	69.5	66.1 ± 4.2	56.31	75.9	61.8 ± 2.2	56.7	67	68.4 ± 4.7	57.4	79.4	64.1 ± 3.6	55.6	72.5	68.1 ± 5.7	54.8	81.4
	SHAM	64.5 ± 3.3	56.9	72.1	68.6 ± 3.4	60.6	76.5	65.3 ± 2.9	58.5	72	65.2 ± 2.8	58.5	71.8	61.3 ± 2.8	54.8	67.8	65.2 ± 1.9	60.6	69.7
	CON	63.5 ± 4.5	53	74	66.7 ± 4.6	56.1	77.4	62.7 ± 4.3	52.7	72.8	66.8 ± 4.4	56.5	77.2	64.1 ± 3.4	56	71.1	66.8 ± 3.3	59.2	74.5
PHR	INT	73.7 ± 4.8	62.5	84.9	73.1 ± 4.5	62.6	83.5	73.8 ± 5.9	60.2	87.5	73.4 ± 6.2	59	87.8	79 ± 4.6	68.3	89.6	74.5 ± 7.5	57.2	91.9
	SHAM	74.6 ± 5.6	61.5	87.7	72.1 ± 4.5	61.6	82.6	73.8 ± 5.2	61.6	86.1	69.4 ± 3.8	66.6	78.2	79.4 ± 6.9	63.5	95.3	76.2 ± 6.96	60.1	92.2
	CON	72.7 ± 8.8	52.2	93.2	77.4 ± 7	61.2	93.6	71.5 ± 8.4	52	91	76.1 ± 7.1	59.6	92.5	75.6 ± 7	59.4	91.8	76.6 ± 5.5	73.8	89.5

The MANOVA test did not detect differences between groups for the RHR after 15 min of *Intervention* between all groups [*W* = 0.446, *F* (2.24) = 0.537, *p* = 0.934, *ηp*^2^ = 0.331]. For this variable, in this moment, univariate ANOVA results for RHR post-day 1 (RHRP1) [*F* (2.24) = 0.103, *p* = 0.903, *ηp*^2^ = 0.008], RHRP2 [*F* (2.24) = 0.152, *p* = 0.860, *ηp*^2^ = 0.012] and RHRP3 [*F* (2.24) = 0.131, *p* = 0.878, *ηp*^2^ = 0.011].

Differences were not detected for the HR after the first CMJ (HR-CMJ) according to MANOVA [*W* = 0.446, *F* (2.24) = 0.537, *p* = 0.934, *ηp*^2^ = 0.331]. The results of the univariate ANOVA also did not show significant differences according to the following results of HR-CMJ Day 1 (HR-CMJD1) [*F* (2.24) = 0.020, *p* = 0.980, *ηp*^2^ = 0.002], HR-CMJD2 [*F* (2.24) = 0.040, *p* = 0.960, *ηp*^2^ = 0.003], HR-CMJD3 [*F* (2.24) = 0.108, *p* = 0.898, *ηp*^2^ = 0.009].

For the HR assessed after the *Intervention* (HR-CMJI), the MANOVA [*W* = 0.446, *F* (2.24) = 0.537, *p* = 0.934, *ηp*^2^ = 0.331] did not detect differences between groups. For the same variable and moment, univariate ANOVA was conducted, HR-CMJI Day 1 (HR-CMJID1) [*F* (2.24) = 0.266, *p* = 0.769, *ηp*^2^ = 0.022], HR-CMJID2 [*F* (2.24) = 0.323, *p* = 0.727, *ηp*^2^ = 0.026], HR-CMJID3 [*F* (2.24) = 0.027, *p* = 0.973, *ηp*^2^ = 0.002], no difference was detected.

The Visual Analogic Scale (VAS) results are described in Table 2. The MANOVA [*W* = 0.027, *F* (2.24) = 1.991, *p* = 0.084, *ηp*^2^ = 0.837] did not detect any differences between the groups. As well as the univariate ANOVA at rest on Day 1 (RVASD1) [*F* (2.24) = 0.749, *p* =, *ηp*^2^ = 0.059]; RVASD2 [*F* (2.24) = 2.648, *p* = 0.091, *ηp*^2^ = 0.181]; and RVASD3 (RVAS-day 3) [*F* (2.24) = 2.615, *p* = 0.094, *ηp*^2^ = 0.179]. The same situation occurred after the *Intervention* (PVAS), as seen on Day 1 (PVASD1) [*F* (2.24) = 0.106, *p* = 0.900, *ηp*^22^ = 0.009]; Day 2 PVASD2 [*F* (2.24) = 1.329, *p* = 0.283, *ηp*^2^ = 0.100]; and PVASD3 [*F* (2.24) = 0.564, *p* = 0.576, *ηp*^2^ = 0.045].

**Table 2:** VAS, visual analog scale; RVAS, rest visual analog scale; PVAS, post-intervention visual analog scale; CI 95%, confidence interval in 95%; M, mean; SD, standard deviation; IL, inferior limit; SL, superior limit.

VAS	GROUP	DAY 1			DAY 2			DAY 3		
		M ± SD	CI (95%)		M ± SD	CI (95%)		M ± SD	CI (95%)	
			IL	SL		IL	SL		IL	SL
RVAS	INT	1.6 ± 0.8	−0.37	3.7	1.7 ± 0.8	−0.1	3.7	2.1 ± 0.6	0.7	3.5
	SHAM	2.1 ± 0.6	0.6	3.6	4.6 ± 1	2.3	7	4 ± 0.3	3.2	4.7
	CON	3 ± 0.7	1.1	4.8	3 ± 0.7	1.2	4.8	3.2 ± 0.7	1.5	4.9
PVAS	INT	1.7 ± 0.8	−0.1	3.7	1.8 ± 0.5	0.5	3.2	2.7 ± 0.5	1.4	4.1
	SHAM	1.4 ± 0.5	0.2	2.6	3.7 ± 1.1	1.3	6.1	1.4 ± 0.4	2.3	4.5
	CON	1.8 ± 0.7	0.1	3.5	2.6 ± 0.7	0.8	4.4	2.6 ± 0.6	1.3	4.1

**Figure 4:**
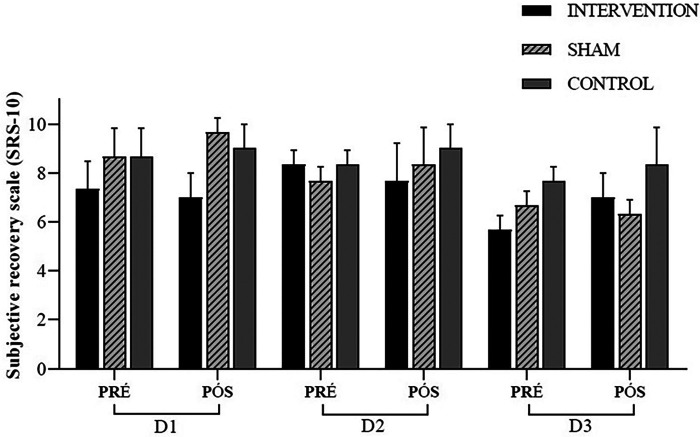
Subjective recovery scale (SRS-10); experimental session—day 1 (D1); experimental session—day 2 (D2); experimental session—day 3 (D3); pre-intervention (pre); post-intervention (post).

For the Subjective Recovery Scale (SRS), the MANOVA results showed no differences [*W* = 0.027, *F* (2.24) = 1.991, *p* = 0.084, *ηp*^2^ = 0.837], as well as the univariate ANOVA in the pre-day 1 (SRSPreD1) [*F* (2.24) = 0.035, *p* = 0.965, *ηp*^2^ = 0.003], SRSPreD2 [*F* (2.24) = 0.000, *p* = 1.000, *ηp*^2^ = 0.000], SRSPreD3 [*F* (2.24) = 2.452, *p* = 0.107, *ηp*^2^ = 0.170], and SRS post-day 1 (SRSPostD1) [*F* (2.24) = 0.304, *p* = 0.740, *ηp*^2^ = 0.025], SRSPostD2 [*F* (2.24) = 0.041, *p* = 0.960, *ηp*^2^ = 0.003], and SRSPostD3 [*F* (2.24) = 1.532, *p* = 0.237, *ηp*^2^ = 0.113] the similarity was maintained (Figure 4).

The one-way ANOVA did not detect variation between all groups at the anthropometric variables. Respectively to the Weight, Height, BMI, and Fat Percentual, the statistical procedure shows the following results [*F* (2.24) = 2.640; *p* = 0.092]; [*F* (2.24) = 1.187; *p* = 0.322]; [*F* (2.24) = 1.156; *p* = 0.332] and [*F* (2.24) = 3.132; *p* = 0.062].

**Figure 5:**
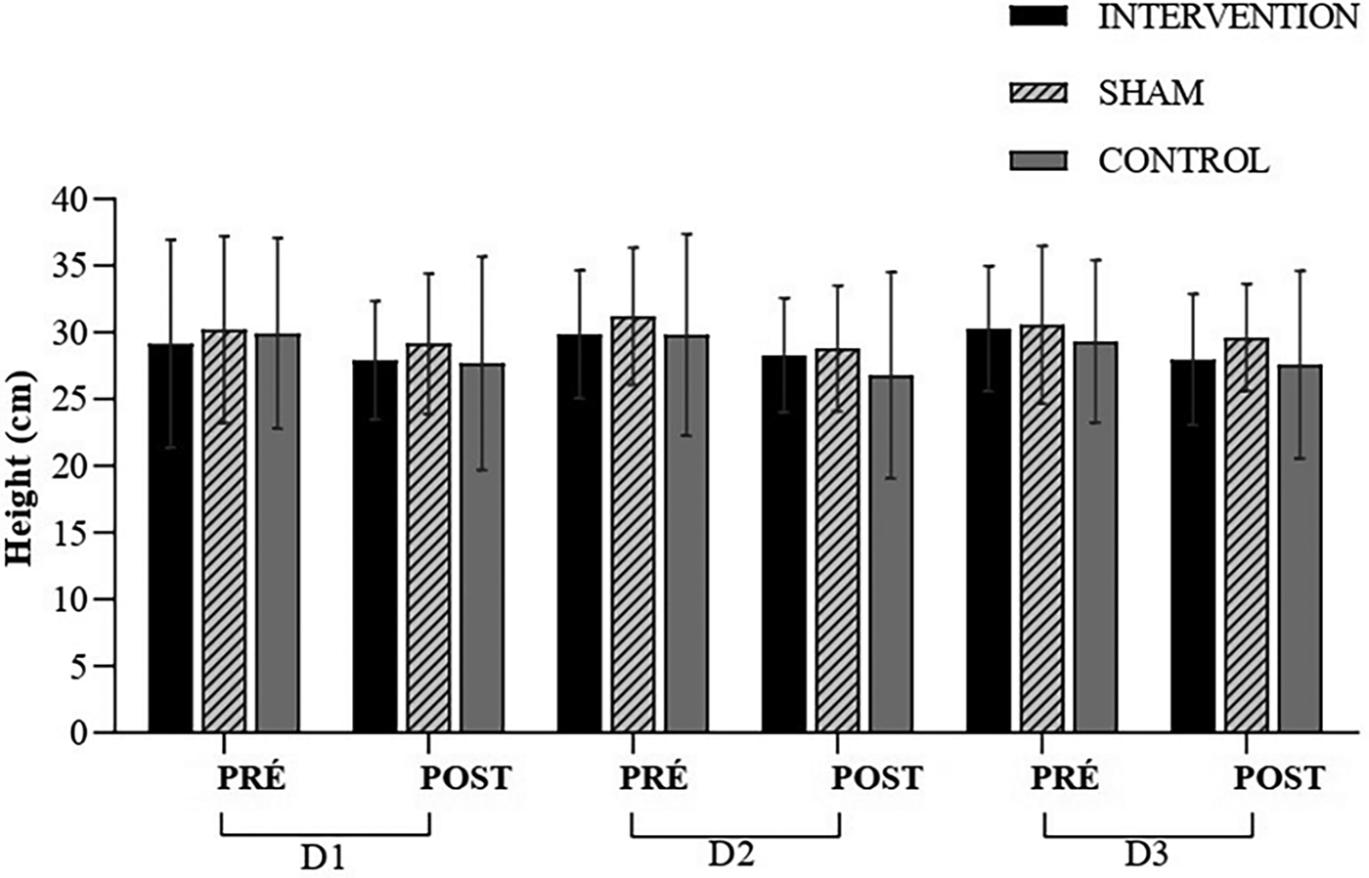
Height (cm); D1; experimental session—day 1 (D1); experimental session—day 2 (D2); experimental session—day 3 (D3); pre-intervention (pre); post-intervention (post).

Thus, for the performance variables, the Jump Height (CMJ-JH) was submitted by the MANOVA test. The result did not detect any significance [*W* = 0.862, *F* (2.24) = 0.244, *p* = 0.994, *ηp*^2^ = 0.071]. Additionally, the one-way ANOVA procedures did not reveal any differences at any time, which was confirmed by the *post hoc* analysis (Figure 5). As seen in the moments Pre Day 1 (CMJ-JHPreD1) [*F* (2.24) = 0.051, *p* = 0.950, *ηp*^2^ = 0.004], Post Day 1 (CMJ-JHPostD1) [*F* (2.24) = 0.155, *p* = 0.857, *ηp*^2^ = 0.013], CMJ-JHPreD2 [*F* (2.24) = 0.161, *p* = 0.852, *ηp*^2^ = 0.013], CMJ-JHPostD2 [*F* (2.24) = 0.294, *p* = 0.748, *ηp*^2^ = 0.024], CMJ-JHPreD3 [*F* (2.24) = 0.124, *p* = 0.884, *ηp*^2^ = 0.010], and CMJ-JHPostD3 [*F* (2.24) = 0.358, *p* = 0.703, *ηp*^2^ = 0.029]. The Table 3 contains results in interactions (time × groups) and time (groups throughout the protocol) according to study variables. The Table 4 contains individual pre and post changes in vertical jump height between groups (Intervention/Sham/Control).

**Table 3:** Results in interactions (time × groups) and time (groups throughout the protocol) according to study variables.

	Interaction			Time		
	*F* [df]	*p*-value	*ηp* ^2^	*F* [df]	*p*-value	*ηp* ^2^
RHR (Bpm)						
Active	3.402 (1–16)^a^	0.084^a^	0.175^a^	1.639 (5–40)	0.172	0.170
Sham	0.362 (1–16)^b^	0.556^b^	0.022^b^	1.470 (5–40)	0.247	0.155
Control	2.093 (1–16)^c^	0.167^c^	0.116^c^	0.815 (5–40)	0.546	0.092
HR (Bpm)						
Active	0.009 (1–16)^a^	0.924^a^	0.001^a^	0.295 (5–40)	0.913	0.036
Sham	0.001 (1–16)^b^	0.976^b^	0.000^b^	0.686 (5–40)	0.522	0.079
Control	0.018 (1–16)^c^	0.894^c^	0.001^c^	0.579 (5–40)	0.716	0.067
VAS (VAS-10)						
Active	0.778 (1–16)^a^	0.391^a^	0.046^a^	0.423 (5–40)	0.830	0.050
Sham	3.879 (1–16)^b^	0.066^b^	0.195^b^	4.137 (5–40)	0.033	0.341
Control	0.009 (1–16)^c^	0.924^c^	0.001^c^	0.862 (5–40)	0.451	0.97
SRS (SRS-10)						
Active	4.445 (1–16)^a^	0.051^a^	0.217^a^	3.067 (5–40)	0.066	0.277
Sham	0.000 (1–16)^b^	1.000^b^	0.000^b^	2.709 (5–40)	0.093	0.253
Control	0.778 (1–16)^c^	0.391^c^	0.395^c^	2.782 (5–40)	0.735	0.510
HCMJ (cm)						
Active	0.102 (1–16)^a^	0.753^a^	0.006^a^	1.173 (5–40)	0.339	0.128
Sham	0.678 (1–16)^b^	0.422^b^	0.041^b^	1.009 (5–40)	0.395	0.112
Control	0.310 (1–16)^c^	0.585^c^	0.019^c^	2.685 (5–40)	0.076	0.251
PCMJ (W)						
Active	0.157 (1–16)^a^	0.698^a^	0.010^a^	1.515 (5–40)	0.207	0.159
Sham	0.123 (1–16)^b^	0.730^b^	0.008^b^	0.585 (5–40)	0.712	0.068
Control	0.010 (1–16)^c^	0.920^c^	0.001^c^	1.231 (5–40)	0.312	0.133
RPE (CR-10)						
Active	0.061 (1–16)^a^	0.809^a^	0.004^a^	0.432 (5–40)	0.823	0.051
Sham	0.119 (1–16)^b^	0.734^b^	0.007^b^	1.105 (5–40)	0.373	0.121
Control	0.729 (1–16)^c^	0.406^c^	0.044^c^	1.205 (5–40)	0.325	0.131

SRS, subjective recovery scale from 0 to 10; VAS, visual analogic scale from 0 to 10; RHR, rest heart rate; HR, heart rate; RPE (CR-10), rating of perceived exertion from 0 to 10; PCMJ (W), vertical jump power in watts; HCMJ (cm), vertical jump height in centimetres; F, Fisher's F; df, degree of freedom; *ηp*^2^, partial eta squared.

^a^ Interaction between Active and Sham group.

^b^ Interaction between Active and Control group.

^c^ Interactions between Sham and Control group.

* Statistical difference.

*p*-value, *p* < 0.05.

**Table 4:** Individual outcomes: CMJ, countermovement jump; Int, intervention; Pre, pre-intervention; Post, post-intervention; GM, group media; SD, standard deviation.

Group	Pré day 1	Post day 1	Pré day 2	Post day 2	Pré day 3	Post day 3	SD
INT	CMJ height (CM)	CMJ height (CM)	CMJ height (CM)	CMJ height (CM)	CMJ height (CM)	CMJ height (CM)	
1	35.43	31.00	34.67	33.27	27.60	31.23	2.62
2	30.10	30.67	30.10	30.67	32.60	25.13	2.28
3	38.27	30.93	38.27	30.93	37.50	33.03	3.27
4	37.93	35.17	31.87	33.00	35.93	37.43	2.20
5	26.63	24.50	29.63	28.67	29.90	26.00	1.99
6	14.63	22.50	24.67	26.17	25.93	25.23	4.01
7	21.47	21.97	23.50	21.30	22.48	21.63	0.75
8	27.00	26.67	25.57	28.07	30.40	26.27	1.57
9	30.90	27.90	30.52	22.50	30.13	25.70	3.02
GM	28.93	27.93	29.78	29.01	30.29	28.25	
SD	8.29	4.73	5.12	3.95	5.02	5.18	
SHAM							
1	31.87	27.87	30.43	26.47	27.83	28.23	1.81
2	29.30	26.33	28.00	24.73	31.23	30.63	2.30
3	28.97	23.90	35.77	27.47	25.43	28.33	3.75
4	30.63	27.73	27.90	27.87	29.27	27.80	1.08
5	39.93	36.20	38.70	36.87	39.32	36.53	1.45
6	41.07	39.47	38.40	36.83	38.70	35.73	1.73
7	27.10	27.20	27.03	26.60	31.97	28.23	1.83
8	24.30	24.56	29.90	24.56	20.60	24.57	2.71
9	18.80	29.27	24.84	27.93	30.87	26.60	3.89
GM	30.22	29.17	31.22	28.81	30.58	29.63	
SD	7.00	5.25	5.12	4.71	5.92	4.03	
CON							
1	31.20	27.50	29.53	21.73	26.63	27.43	2.93
2	30.43	24.40	26.50	22.87	26.50	22.87	2.63
3	25.70	30.97	26.70	24.57	23.57	24.90	2.39
4	39.43	37.77	39.83	40.40	40.50	41.00	1.05
5	37.83	37.63	39.97	34.83	36.23	34.33	1.92
6	26.73	22.57	30.13	26.43	27.17	26.40	2.20
7	34.67	32.66	36.64	33.89	34.63	33.91	1.20
8	35.30	31.70	33.17	31.70	34.23	31.70	1.41
9	16.27	12.37	15.07	14.50	24.30	19.23	3.88
GM	30.84	28.62	30.84	27.88	30.42	29.09	
SD	7.19	8.03	7.83	7.99	6.05	6.73	

**Figure 6:**
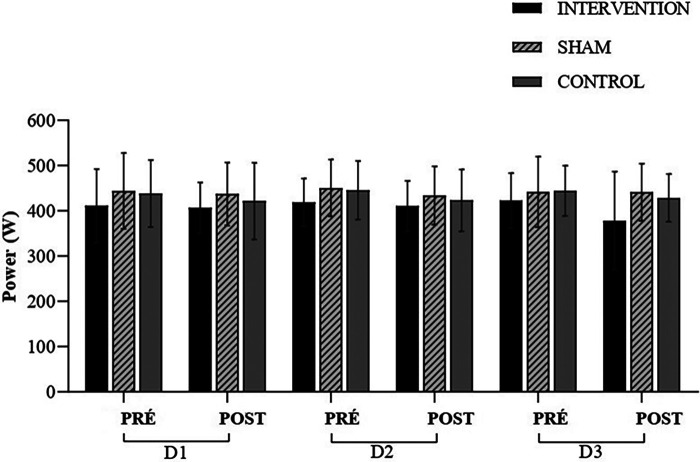
Power (W); experimental session—day 1 (D1); experimental session—day 2 (D2); experimental session—day 3 (D3); pre-intervention (pre); post-intervention (post).

For the Vertical Jump Power (CMJ-PO), the MANOVA results did not detect any differences [*W* = 0.629, *F* (2.24) = 0.827, *p* = 0.623, *ηp*^2^ = 0.207] according to Figure 6. When examining the data in the univariate ANOVA, no differences were observed. These moments are as follows: CMJ-PO Pre Day 1 (CMJ-POPreD1) [*F* (2.24) = 0.435, *p* = 0.652, *ηp*^2^ = 0.035], CMJ-PO Post Day 1 (CMJ-POPostD1) [*F* (2.24) = 0.407, *p* = 0.670, *ηp*^2^ = 0.033], CMJ-POPreD2 [*F* (2.24) = 0.726, *p* = 0.494, *ηp*^2^ = 0.057], CMJ-POPostD2 [*F* (2.24) = 0.310, *p* = 0.736, *ηp*^2^ = 0.025], CMJ-POPreD3 [*F* (2.24) = 0.285, *p* = 0.754, *ηp*^2^ = 0.023], and CMJ-POPostD3 [*F* (2.24) = 1.627, *p* = 0.217, *ηp*^2^ = 0.119].

**Figure 7:**
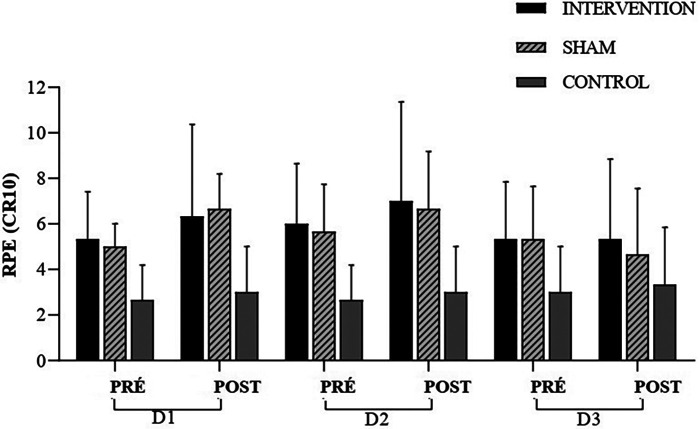
RPE, rating of perceived exertion; experimental session—day 1 (D1); experimental session—day 2 (D2); experimental session—day 3 (D3); pre-intervention (pre); post-intervention (post).

The MANOVA did not detect any differences in Rating of Perceived Exertion (RPE) throughout the protocol [Wilks' Lambda = 0.027, *F* (2.24) = 1.991, *p* = 0.084, *ηp*^2^ = 0.837] (Figure 7). When examining the data in a univariate manner (univariate ANOVA), no differences were observed. These moments are as follows: moment Pre Day 1 (RPEPreD1) [*F* (2.24) = 0.225, *p* = 0.801, *ηp*^2^ = 0.018], Post Day 1 (RPEPostD1) [*F* (2.24) = 0.695, *p* = 0.509, *ηp*^2^ = 0.055], RPEPreD2 [*F* (2.24) = 0.626, *p* = 0.543, *ηp*^2^ = 0.050], and at RPEPostD2 [*F* (2.24) = 1.109, *p* = 0.346, *ηp*^2^ = 0.085], RPEPreD3 [*F* (2.24) = 0.960, *p* = 0.397, *ηp*^2^ = 0.074], and RPEPostD3 [*F* (2.24) = 0.304, *p* = 0.740, *ηp*^2^ = 0.025], the similarity was maintained.

## Discussion

The present study investigated the effect of transcranial direct current stimulation on CMJ in soccer players. Some studies show that the tDCS technique can influence physical/sports performance (17, 26). However, according to the results presented related to the tDCS montage contrary to our hypothesis, tDCS did not influence performance on the CMJ and perception of effort and recovery. These results are in line with previous studies that did not report significant improvements in performance (46, 47). However, other studies showed positive effects of tDCS on performance (48, 49).

The tDCS has been considered a technique influencing different physical/sports performance aspects. However, studies conducted with athletes involving specific tasks are scarce, and the heterogeneity of the adopted protocols does not allow for any conclusions about its ergogenic effects (19).

In terms of performance variables, several ergogenic measures aimed at optimizing physical performance, attenuating fatigue mechanisms, and facilitating post-physical exertion recovery have been widely studied (50–52). For example, caffeine (53) has shown improvements in vertical jump performance in soccer players, while in another study (54), caffeine ingestion was found to improve tactical performance in soccer athletes. As for tDCS used as an ergogenic measure to improve performance, Lattari et al. (23) demonstrated that anodal tDCS (2 mA for 20 min, with the anode positioned over the Cz point and the cathode over the right supraorbital area) led to improved jump performance in individuals considered advanced in strength training. However, unlike the individuals Lattari et al. (23) investigated, the individuals included in the present study are frequently exposed to CMJ as part of training load control. Therefore, they are likely better trained in the task, presenting a lower potential for improvement.

Regarding the subjective variables, a study by Moreira et al. (26) demonstrated improvement in the Well-Being Questionnaire of soccer players after 20 min of tDCS was applied in the Left Dorsolateral Prefrontal Cortex (DLPC) on the day following an official match. In another study by Valenzuela et al. (32) with swimmers, an improvement in the RPE was observed. In contrast to the present study's hypothesis, anodic tDCS applied to the M1 did not influence the responses of subjective scales in soccer players. It is worth noting that the stimulated area in the cited articles differs from the present study. Although it is not clear how the DLPFC and M1 are connected, a recent study reported that their excitabilities are linked (55). These findings agree with previous studies that also failed to show a reduction in RPE or an increase in performance after the application of tDCS over (20, 56).

Subjective assessments are recommended to monitor the psychometric recovery status of football players to detect early signs of fatigue and optimize high-level training performance (57). The decrease in these measurements are used to express the lesser subjective state of fatigue, effort and recovery of a player or team during training or competition. Improvements in these scores are significant predictors of performance in football athletes (58, 59). Likewise, insufficient recovery, poor quality of sleep from the previous night and increased levels of stress, fatigue (60, 61) during training can negatively impact athletic performance (62, 63). Thus, monitoring these psychometric measures (i.e., through subjective scales) in football players can assist coaches in programming and appropriately adapting training loads in order to maximize performance and reduce the risk of injuries, overtraining and overreaching functional (64–66).

Machado et al. (19) demonstrated that this topic still has limitations, mainly related to the type of montage, stimulation duration, the task performed, and athlete's level.

According to the established montage, performance was sometimes even better for the SHAM group. Therefore, the proposed montage in this study (CZ + Inion) did not seem to be effective for this Intervention, unlike classic montages such as the motor cortex and LDPC (M1 + Fp2) (67) or only the M1 (C3, and C4) (68), among other montages presented by Alix-Fages (20) and Machado (19).

However, this study was conducted in only three sessions, representing acute effects. Therefore, conducting further studies with this montage in a more prolonged intervention approach (chronic effect) would be interesting.

Nevertheless, this is the first study to evaluate the CMJ performance in soccer players throughout the competition period in soccer players. It also is the first study with this tDCS montage in the sports literature. Therefore, this article opens new possibilities for studying the essential effects of various ergogenic measures related to tDCS or other techniques, such as transcranial magnetic stimulation (TMS), as well as different montage configurations and performance variables in soccer or other specificities.

## Conclusion

The results the present study showed that transcranial direct current stimulation (tDCS) in the CZ area at the M1 cortex for 15 min, in three sessions, did not improve vertical jump in the CMJ performance and did not improve the results at the subjective scales in soccer players. Future research should explore the effects of tDCS on the performance of professional football athletes. Furthermore, different tDCS configurations such as intensity, sessions, areas (e.i) should be explored in order to find optimal tDCS protocols to improve performance in different physical conditions. The application of tDCS in the setting of this study is not recommended to increase vertical jump performance or reduce perceived exertion and recovery.

## Limitations

There are limitations in the present study besides the prolonged stimulation mentioned above. First, this study should have included measurements to explain the neurophysiological effects of tDCS. Future studies should employ measures to assess neurophysiological effects, such as electromyography and electroencephalography, among others, at the target muscle areas. Additionally, the players were not separated by position, which may result in differences in CMJ performance, as specific position-specific requirements may involve more jumping, such as goalkeepers or defenders.

## Data Availability

The original contributions presented in the study are included in the article/Supplementary Material, further inquiries can be directed to the corresponding author.
